# A mixed methods evaluation of the impact of ECHO^®^ telementoring model for capacity building of community health workers in India

**DOI:** 10.1186/s12960-024-00907-y

**Published:** 2024-04-23

**Authors:** Rajmohan Panda, Supriya Lahoti, Nivedita Mishra, Rajath R. Prabhu, Kalpana Singh, Apoorva Karan Rai, Kumud Rai

**Affiliations:** 1 Public Health Foundation of India (PHFI), Gurugram, Haryana India; 2 Extension for Community Healthcare Outcomes (ECHO) India, Okhla Phase III, New Delhi, India; 3 HexaHealth, Gurugram, Haryana India; 4 Hamad Medical Corporation, Doha, Qatar

**Keywords:** Community health workers (CHWs), Accredited social health activists (ASHAs), Maternal and child health, Primary healthcare, Health worker training, ECHO telementoring, Mixed-method study

## Abstract

**Introduction:**

India has the largest cohort of community health workers with one million Accredited Social Health Activists (ASHAs). ASHAs play vital role in providing health education and promoting accessible health care services in the community. Despite their potential to improve the health status of people, they remain largely underutilized because of their limited knowledge and skills. Considering this gap, Extension for Community Healthcare Outcomes (ECHO)^®^ India, in collaboration with the National Health System Resource Centre (NHSRC), implemented a 15-h (over 6 months) refresher training for ASHAs using a telementoring interface. The present study intends to assess the impact of the training program for improving the knowledge and skills of ASHA workers.

**Methods:**

We conducted a pre–post quasi-experimental study using a convergent parallel mixed-method approach. The quantitative survey (*n* = 490) assessed learning competence, performance, and satisfaction of the ASHAs. In addition to the above, in-depth interviews with ASHAs (*n* = 12) and key informant interviews with other stakeholders (*n* = 9) examined the experience and practical applications of the training. Inferences from the quantitative and qualitative approaches were integrated during the reporting stage and presented using an adapted Moore’s Expanded Outcomes Framework.

**Results:**

There was a statistically significant improvement in learning (*p =* 0.038) and competence (*p =* 0.01) after attending the training. Participants were satisfied with the opportunity provided by the teleECHO™ sessions to upgrade their knowledge. However, internet connectivity, duration and number of participants in the sessions were identified as areas that needed improvement for future training programs. An improvement in confidence to communicate more effectively with the community was reported. Positive changes in the attitudes of ASHAs towards patient and community members were also reported after attending the training. The peer-to-peer learning through case-based discussion approach helped ensure that the training was relevant to the needs and work of the ASHAs.

**Conclusions:**

The ECHO Model ™ was found effective in improving and updating the knowledge and skills of ASHAs across different geographies in India. Efforts directed towards knowledge upgradation of ASHAs are crucial for strengthening the health system at the community level. The findings of this study can be used to guide future training programs.

**Trial registration** The study has been registered at the Clinical Trials Registry, India (CTRI/2021/10/037189) dated 08/10/2021.

**Supplementary Information:**

The online version contains supplementary material available at 10.1186/s12960-024-00907-y.

## Introduction

The Alma Ata Declaration of 1978 has recognized primary health care as an essential element for improving community health. Community health workers (CHWs) have the potential to complement an overstrained health workforce and enhance primary healthcare access and quality [1]. Low- and middle-income countries (LMICs) face a triple burden of low density of doctors and nurse-midwives, low government expenditure on health, and disproportionately larger poor health outcomes [2]. The roles and responsibilities of CHWs vary across LMICs [3]. A systematic review has documented that the socio-cultural, economic, health system, and political context in which CHW interventions operate in LMICs influence the implementation and success of interventions [4].

The National Rural Health Mission (NRHM), India introduced Accredited Social Health Activists (ASHAs) as female CHWs in 2005. The ASHAs are women volunteers selected from the local village and were initially conceptualized with a vision to improve maternal and child health in the country; however, over time, they are now involved in different national health programmes [5, 6]. Despite their potential to contribute to preventive and promotive healthcare, they remain largely underutilized because of their limited knowledge and skills [1]. The World Health Organisation (WHO) has suggested ‘regular training and supervision’ for CHWs to fulfil their role successfully [7]. In India, the health system lacks methods for continuous education and routine upgradation of the ASHA’s skills [8–10].

In LMICs, digital training programs can help expand the reach of training to large numbers of healthcare workers at a low cost without interfering with the delivery of routine healthcare services [11, 12]. An evidence-mapping study of 88 studies that used technology for training CHWs in LMICs found that the focus of trainings was maternal and child health and other high-burden diseases were neglected [13]. In India, studies evaluating digital trainings for CHWs have focussed on specific diseases or have been limited to specific states in the last decade [10, 14]. This study was conducted across multiple states. More such studies with larger sample size are needed on the evaluation of such training initiatives in India [13, 15, 16].

Project Extension for Community Healthcare Outcomes (ECHO) presents an educational opportunity for capacity-building through a telementoring platform that uses video conferencing to create a continuous loop of learning and peer support. The sessions are facilitated by didactic presentation and case-based learning that allows problem-solving through shared best practices [17]. ECHO India, in collaboration with National Health System Resource Centre (NHSRC), provided refresher training for ASHAs [18]. There is increasing evidence of the positive effect of ECHO training on medical provider’s learning and self-efficacy. However, its value as a training platform to CHWs in LMICs is limited. Previous studies that evaluated the use of the ECHO Model ™ for CHWs focussed on specific diseases and were conducted in high-income countries (HICs) [19–21]. For the adoption of digital technology, CHWs in LMICs encounter challenges such as poor proficiency levels in accessing and using digital platforms, limited access to troubleshooting, poor internet connectivity, and in-house support for resolving issues [22]. The present study was designed to assess the impact of the ECHO telementoring model for improving the knowledge and skills of ASHA workers in delivering comprehensive health services. This will provide new insights for measuring outcomes of digital training programs for CHWs (ASHA workers).

## Methods

### Study design

A pre–post quasi-experimental design using a convergent parallel mixed-method approach [23] was employed. The quantitative and qualitative data were collected concurrently. Inferences from both approaches were integrated during the reporting stage. This allowed for a comprehensive understanding of the effect of training on the knowledge and skills of ASHAs.

### The ECHO training intervention and curriculum

Project ECHO^®^ designed a 15-h (over 6 months from October 2021 to March 2022), virtual, refresher training program to enhance the capacity of ASHAs to deliver counselling services for comprehensive healthcare in four states (*n* = 2293). Each session lasted for 90 min. The ECHO NHSRC training used a “hub and spoke” structure in which a multidisciplinary team of experts (trainers) based at a regional academic medical centre (the “hub”) engaged with the ASHAs (the “spokes”) [24] who attended the sessions from dedicated learning sites (PHCs). Each site also had a coordinator who would help facilitate the discussions and questions. The training curriculum was developed based on the NHSRC ‘ASHA training modules’ [18] in the regional languages in consultation with partners (hub-leaders and trainers). It comprised 10 sessions covering a range of topics, such as maternal health, new-born care, child health, nutrition, reproductive health, violence against women, tuberculosis, vector-borne diseases, non-communicable diseases, COVID-19, palliative care, and mental health. The training presentations included text with visual learning methods, such as images, videos, and links to training resources.

### Study settings

The evaluation study was conducted in four states of India, where training sessions were held. These states represented the four geographical regions—northern (Himachal Pradesh) (*n =* 499), southern (Tamil Nadu) (*n =* 500), eastern (West Bengal) (*n =* 618), and north-eastern (Sikkim) (*n =* 676). The intervention (training sessions) was completed in March 2022. The end-point data were collected from March 2022 to May 2022.

### Study participants and recruitment

Simple random sampling was used to select the ASHAs from each state for the quantitative survey. The participants were recruited from a list of ASHAs who would be receiving the ECHO NHSRC training. To be included, ASHAs had to be enrolled in the refresher training, planning to continue working for the next 10 months, with available contact details and consenting voluntarily. The ASHAs were contacted through mobile phones in each state. Key informant interviews (KIIs) were conducted with hub leaders who were involved in implementing the training, trainers (faculty) who delivered the lectures, and in-depth interviews (IDIs) with ASHAs.

### Sample size

The sample size for the quantitative study was estimated by assuming a 25% improvement in knowledge and skills, 80% power, and a design effect factor of 1.7%. An adjustment of 30% loss to follow up and 20% non-response (from previous experience) led to a sample of 591 participants across four states, i.e., 148 participants from each state. For the qualitative study, purposive sampling with maximum variation across age, education, practice sites, and years of work experience was used for the selection of the participants. A total of 12 IDIs were conducted with ASHAs and nine KIIs with stakeholders (Additional file 2: Appendix S2).

### Study tools and data collection

For quantitative data collection, a structured questionnaire was designed through a collaborative approach with the research and program implementation team. The knowledge of ASHAs was assessed by a combination of 18 technical questions and case vignettes. Learning and competence, performance, and satisfaction were assessed with a 5-point Likert scale, using 1 = Strongly Disagree; 2 = Disagree; 3 = Neither Agree nor Disagree; 4 = Agree; and 5 = Strongly Agree. The face validity of the questionnaire was tested with ten ASHAs, separate from those recruited in the study and five primary care experts. The changes related to language, clarity, and relevance were made in the questionnaire based on the feedback from experts and participants. Separate discussion guides were developed for KIIs with trainers (Additional file 3: Appendix S3) and hub-leaders (Additional file 4: Appendix S4) and IDIs with ASHAs (Additional file 5: Appendix S5). The guide focussed on examining the experience and practical applications of the training and was field tested before being administered in the main study. All study tools were translated into the local languages of the states and back-translated to check discrepancies.

The data were collected on the cell phone by experienced and trained researchers from social sciences backgrounds. Due to telephonic data collection, we were unable to capture non-verbal interview data such as emotions or gestures, particularly important in qualitative data. This may affect the richness of data and interpretation of responses. The quantitative tool was designed in the CS Pro software (version 7.5) and data were collected using its smartphone application. The qualitative interviews lasted around 40–50 min and were audio recorded. All interviews were translated and transcribed verbatim.

### Data analysis

We summarized the quantitative data using descriptive statistics. Continuous variables were summarized using mean ± SD, and categorical variables were summarized using percentages and frequencies. The responses recorded using the 5-point Likert scale were recategorized during the analysis into three categories, i.e., ‘agree’ (combining strongly agree and agree), ‘disagree’ (combining strongly disagree and disagree), and ‘neutral [25]. Paired *t* test was used to find the difference between the pre- and post-scores of learning and competence and the attitude of participants toward ECHO training. McNemar’s test was used to assess changes in pre- and post-test scores for the technical domain. A *p* value of less than 0.05 was considered significant. STATA 16.0 statistical software was used for the analysis.

Qualitative data were analyzed according to the principles of the Framework approach [26], which combines inductive and deductive approaches. As a first step, two authors (SL and NM) familiarized themselves with four randomly selected transcripts and independently coded them using initial codes that were developed based on Moore’s framework levels of participation, satisfaction, learning, competence, and performance [27]. New codes that emerged while undertaking the analysis were included. The discussion and comparison of the double-coded transcripts enabled the development of an agreed set of codes. Any disagreements were discussed and resolved with the help of the third author (RP) to achieve inter-coder agreement. A final codebook was developed and applied to all the transcripts. The codes were combined and categorized into key emerging themes., The themes, including quotes (respondents’ exact words), were included to represent the main findings. Atlas.ti (version 8) software was used for data analysis.

## Results

### Moore’s level 1—participation

Table 1 represents the baseline demographics of the recruited participants. From 610 participants who completed the pre-training survey, 490 participants completed the post-training survey, resulting in a follow-up rate of 80% (95% CI 76.6, 83.1). A total of 120 (20%, 95% CI 16.8, 23.3) participants were lost to follow up. This was due to a) contact numbers not being operational (*n =* 96) and b) refusal due to time considerations (*n =* 24). The field investigators attempted three additional phone calls, coordinated with hubs for participants’ alternate contact information, and offered flexible phone appointments to ensure maximum participation in the post-training survey. The majority (68%) of ASHAs were posted at sub-centres. A sub-centre is the most peripheral unit of contact of the health system with the community [28]. The majority of the participants (75%) had completed their high school (10th) education.

**Table 1:** Demographics of the study participants

Demographic characteristics	*n* (%)
Age^a^ (mean ± SD)	38.2 (7.9)
Site of posting	
Sub centre	417 (68.4%)
Primary health centre	193 (31.6%)
Education qualification	
Primary (5th)	26 (4.3%)
Secondary (8th)	126 (20.7%)
High school (10th)	209 (34.3%)
Higher secondary (12th)	147 (24.1%)
Graduation and above	102 (16.7%)
Professional years of experience^a^ (mean ± SD)	7.8 (5.7)
Type of mobile phone	
Smartphone	502 (82.3%)
Qwerty phone	108 (17.7%)

^a^ In completed years

A hub leader described the efforts made by the ECHO to facilitate the participation of the ASHAs in the training.

“ECHO provided a facility where everyone can gather at the nearest block for the training. Physical and online modes [are] both available” (Hub-leader, Himachal Pradesh).

### Moore’s level 2—satisfaction

The end-point survey assessed participants’ satisfaction with the ECHO training. The survey included eight items that measured overall training satisfaction and five items that measured satisfaction with factors specific to the telementoring model using close-ended questions. Satisfaction with the training content and environment was measured with four items. Except for one topic area (sharing of additional resources and training material), over 90% of participants were satisfied with almost all of the different components of the ECHO telementoring intervention (Additional file 1: Appendix S1, Tables S1.1, S1.2, S1.3). While participants found the overall intervention favourable, 54.5% of all participants were dissatisfied with internet connectivity in the training sessions. Around one fourth of the participants faced challenges with the duration (31.2%), frequency (31.2%), and number of participants (28.4%) in the sessions (Additional file 1: Appendix S1: Table S1.3).

The qualitative findings also show that most of the trainees were satisfied with the learning opportunity provided by the ECHO training.

“After attending these ECHO sessions, I felt we are constantly learning new techniques and it’s a deep sense of satisfaction” (ASHA, Tamil Nadu).

The ASHAs also shared areas or features of the ECHO model that did not meet their requirements and need improvement. They felt that the duration allotted for a session was not sufficient and some topics were covered very fast.

“They rush a lot while teaching over phone. It will be more helpful if they take more time and explain the things in a more detailed manner” (ASHA, WB)

Another ASHA suggested increasing the duration of training to improve their understanding of some topics.

"Increase the time of the training. Topics can be made deeper, and richer for better explanations" (ASHA, Tamil Nadu)

ASHAs described challenges related to connectivity while attending the training.

“The network connection was a problem and video used to lag” (ASHA, Sikkim)

Trainers shared their opinion about aspects of online trainings that did not meet their expectations.

“The problem is that they only join the meeting [online training] and do their own work, they actually do not listen properly.” (Trainer, WB)

A trainer mentioned that the large number of participants in some sessions affected the interaction among participant ASHAs.

“Sometimes a session has too many participants causing coordination efforts to be a challenge in these sessions” (Trainer, TN)

Difficulties in reaching the PHCs were recorded from the state of Sikkim. The geographical location and lack of transport facilities were mentioned by a trainer.

“We have transportation problem, our ASHA comes from rural area and it’s difficult to get taxi, which makes [it] harder to attend classes” (Trainer, Sikkim)

Many participants regarded organizational support as a facilitator for attending the training program. An ASHA from Tamil Nadu described how the issue of distance was resolved through management interventions from the organization.

“Our Block is 30 km away. There is another Block nearby that is 1 km only from here, they sent us there… so there was no problem” (ASHA, TN)

### Moore’s level 3—learning

**Fig. 1:**
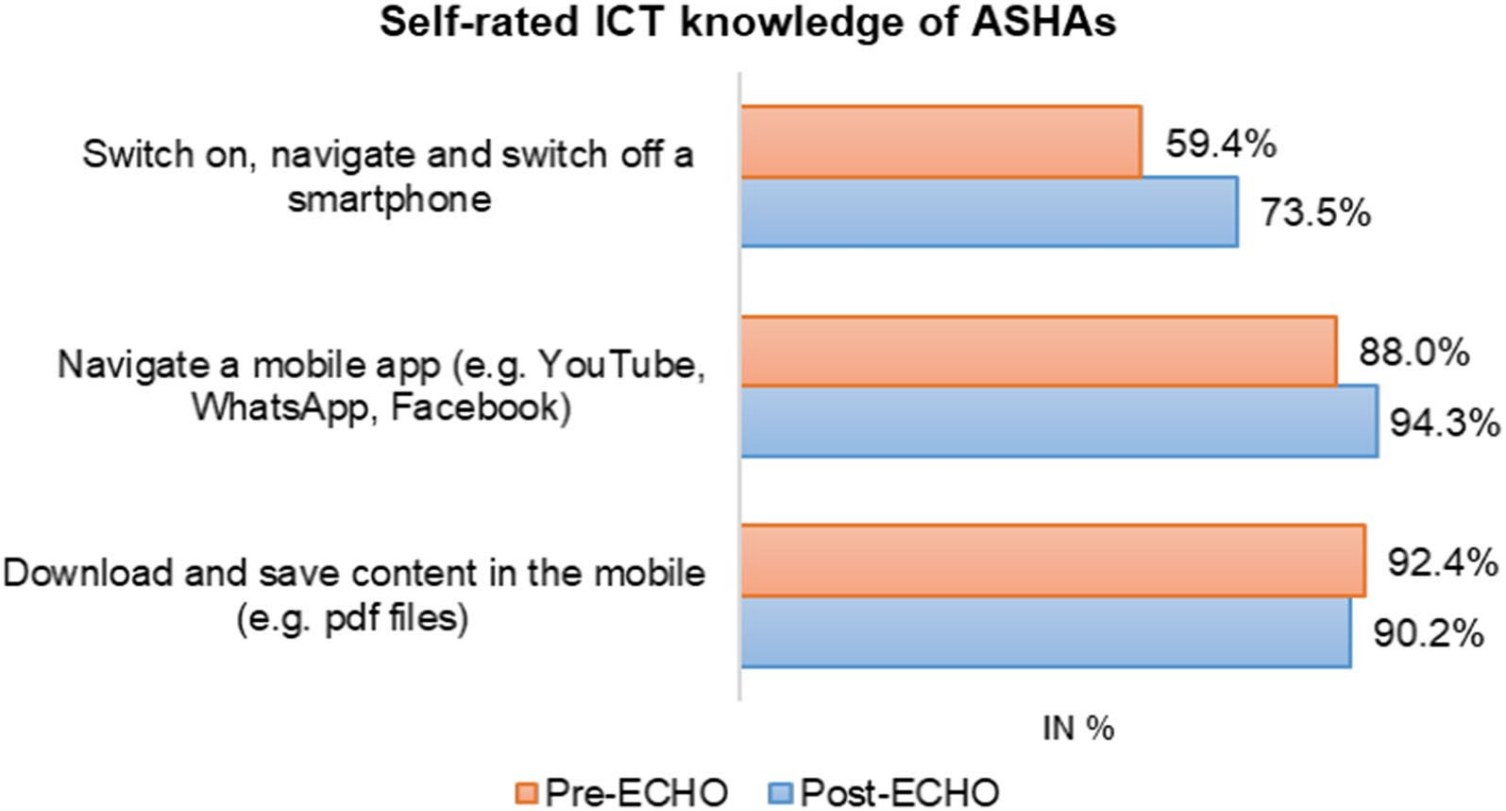
Self-rated ICT knowledge of ASHAs

McNemar’s Chi-square statistic showed a significant difference between pre-ECHO and post-ECHO proportions in various aspects of health-related technical knowledge. Before the training, 1% of participants were aware of the correct schedule to be followed in the first week after the delivery of a child, which increased to 40% of participants post-training (p < 0.001). Overall, a statistically significant increase of 6% (95% CI 0.0003, 0.12; *p =* 0.038) in participants’ technical knowledge after ECHO training was found. After the training, a 7% increase in knowledge of malaria (*p =* 0.002) and its symptoms and a 9% increase in knowledge of the right action to be undertaken (p < 0.001) was reported. Knowledge related to some areas such as recommended duration of physical activity or exercise (p < 0.001), immunisation after child birth (*p =* 0.001), family planning in women after child birth (*p =* 0.002) showed a decrease after attending the training (Additional file 1: Appendix S1, Table S2). Post ECHO training, ASHAs reported an improvement in their knowledge of using a smartphone (switch on and off, and navigate) (*p =* 0.0005) and navigating a mobile application (*p =* 0.59). The ASHAs reported a 2% decrease in their knowledge of downloading content in the mobile (*p =* 0.07) (Fig. 1).

The qualitative data show that ASHAs who did not have a smartphone found it difficult to download and save content. One of the participants reported receiving additional training content in the form of a pdf file. She also mentioned that those who do not use a smartphone find it challenging to access this additional resource.

“We get the study material in a pdf so that simplifies our work further. But those who do not have a smartphone, find it difficult to get this opportunity” (ASHA, WB)

#### 3A—Declarative learning

Declarative learning assesses how participants articulate the knowledge that the educational activity intended them to know (knowing what). The qualitative findings show that the training had increased the ASHA’s knowledge in specific domains such as breastfeeding during COVID-19.

“The doubt was whether a mother can breastfeed the baby when suffering from COVID-19. I got clarity about that… many such topics were cleared” (ASHA, Himachal Pradesh)

#### 3B—Procedural learning

Procedural learning assesses the participants' articulation of how to do what the educational activity intended them to know (knowing how).

Participants reported that they had gained new skills related to the approach and identification of healthcare issues after attending the ECHO training.

“Earlier we wouldn’t know if ear related issues had a resolution – But following the ear related training we are aware that such issues can be cured or have treatments” (ASHA, Tamil Nadu).

The qualitative interviews revealed additional themes that described the value of the ECHO training program in improving the learning experience of ASHAs.

ASHA workers felt that the case presentations from their peers enhanced their learning experience.

“One ASHA shared a case of an anaemic mother. Based on this case we learned that this could have been prevented if iron tablets are provided from the adolescent stage” (ASHA, Tamil Nadu).

The interactive nature of the sessions and the discussions benefitted the learning experience of the ASHAs.

“Open discussion helped us so much. We can discuss any topics if we haven’t understood and sir used to explain again” (ASHA, Sikkim)

#### Moore’s level 4—competence

The participants reported significant improvement in their confidence to identify and manage several health conditions like birth asphyxia (for home deliveries) and management with a mucus extractor (*p =* 0.01), screen and refer pregnant women (*p =* 0.01), disseminate information on domestic violence and sexual harassment (*p =* 0.001). Overall, a statistically significant increase of 6% (95% CI 0.01, 0.10; *p =* 0.01) in participants’ competence after attending the ECHO training was found. Participants reported a decrease in their confidence to track child immunisation (*p* < 0.001), monitor symptoms of COVID (p < 0.001), and clarify concerns of the community (*p* < 0.001) after attending the training (Additional file 1: Appendix S1, Table S3).

Participants mentioned an improvement in their confidence while communicating with patients and their families.

“Initially we could not talk to people so comfortably, we hesitated at times but after being trained we can talk to people and their families properly and easily now” (ASHA, West Bengal)

An ASHA described a gap in their ability to talk to mothers in the field and suggested including more training content on efficient communication skills.

“We go on field and talk to mothers. There was no training for these, but I feel it will be good if we can have training on how to talk to mothers comfortably” (ASHA, WB)

#### Moore’s level 5—performance

**Fig. 2:**
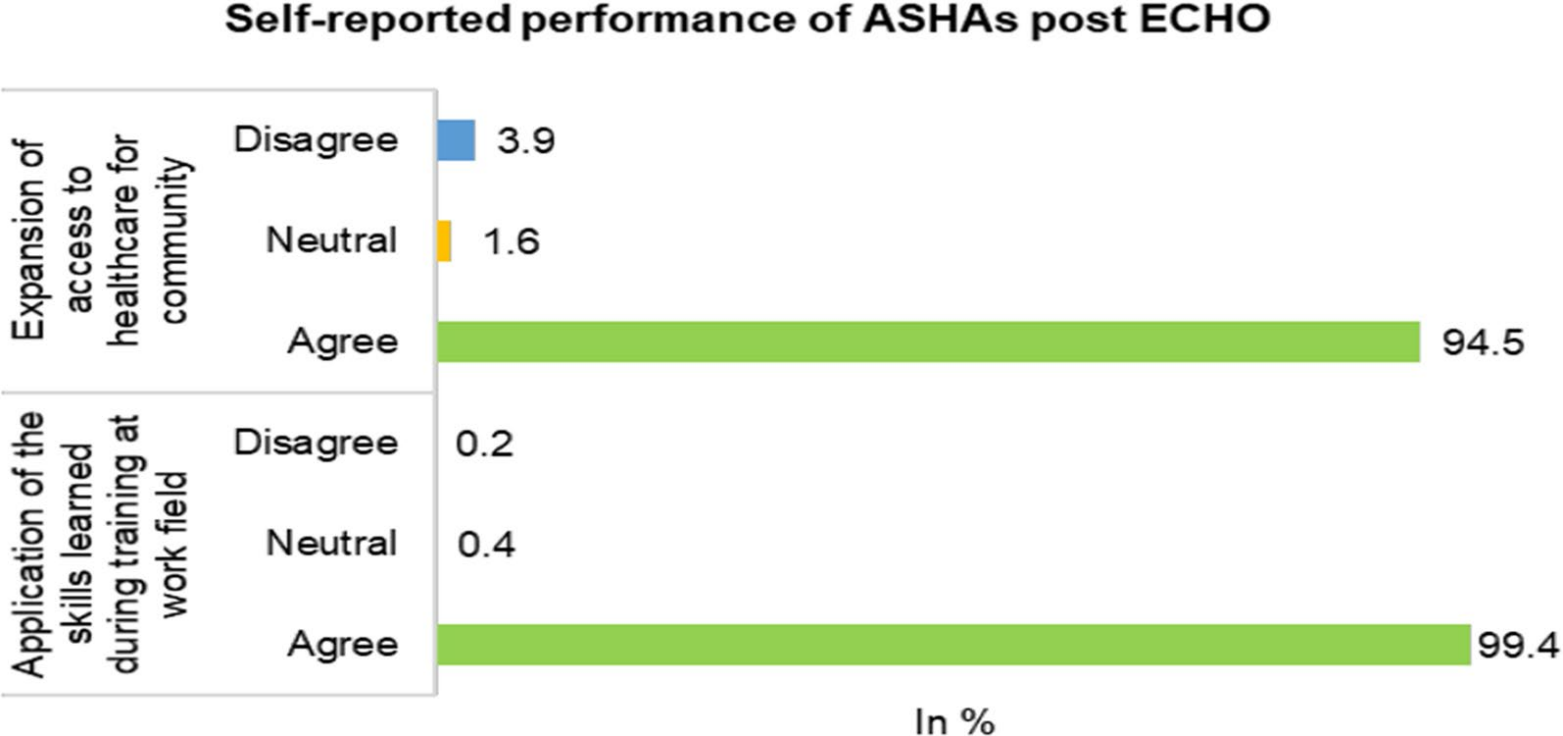
Self-reported performance of ASHAs

The study identified a significant improvement in ASHAs’ positive attitude toward maternal and child health issues. Overall, a 5% improvement (95% CI − 0.009, 0.10; p value = 0.09) in participants’ attitudes post-ECHO training was found. Almost all the participants (99%) reported applying the skills learnt during the training at their workplaces. More than 90% of the participants felt that the ECHO training expanded access to healthcare in their community (Fig. 2). The ASHAs reported an improvement in their attitude towards inclusion of HIV patients in the community (*p =* 0.01) and home visits for new born babies (p < 0.001) (Additional file 1: Appendix S1, Table S4).

The ASHAs shared specific examples where they made changes in their practice or treatment strategies after attending the training.

“[Earlier] the implementation was not proper [correct]. As an example, if a child’s life has to be saved on the spot, we would take the medicines and syringes separately. Now we take the necessary items section wise including the AFI kit. So that’s the change” (ASHA, Tamil Nadu).

## Discussion

The results of this evaluation suggest that Project ECHO provides a suitable and efficacious platform for training for ASHAs. The participants reported an improvement in their knowledge, skills, and practices. They also described improved confidence to communicate more effectively. Some areas in which the ASHAs reported a lack of knowledge and confidence include newborn immunisation and family planning after pregnancy.

The NRHM guidelines for the recruitment of ASHAs require candidates to have at least eight or 10 completed years of formal education. Low literacy and inadequate training of ASHAs have been observed in different states in India [30, 31]. However, with the proper training and support, ASHAs can provide comprehensive preventive and promotive healthcare services [29]. In this study, the majority (75%) of ASHAs across all states had ten or more years of schooling. The ECHO training will bolster their knowledge, skills, and confidence in providing effective services.

The ASHAs receive 23 days of training in the first year, followed by 12 days of training in every subsequent year to keep them updated with the knowledge and skills needed to effectively perform their roles and responsibilities. Previous studies have identified many challenges in the training of ASHAs, such as lack of regular refresher training [32], shortage of competent trainers, insufficient funds, and use of obsolete health information [33]. The training programs have mostly been didactic-based and had limitations in the engagement of participants [34]. The ECHO NHSRC refresher training addresses these limitations by promoting peer-to-peer learning and through a case-based discussion approach [35].

Our findings report a significant increase in the knowledge of ASHA workers with respect to specific domains like maternal and child health. A randomized controlled trial in Karnataka, India, found a significant improvement in mental health knowledge, attitude, and practice (KAP) scores amongst ASHAs trained by a hybrid training (traditional 1-day in-person classroom training and seven online sessions using the ECHO Model) against conventional classroom training [14]. This study findings highlight the improvement in knowledge of ASHAs related to oral health and palliative care post-ECHO training. An improvement in knowledge has also been observed in other studies that have evaluated ECHO telementoring interventions in cancer screening [36], palliative care [37, 38], HIV [39], and chronic pain [40] In this study, ASHAs reported poor knowledge of the immunisation schedule for a newborn as well as the confidence to record and track immunisation in the community even after the ECHO training. A critical function of ASHAs is to assist ANMs or nurses with all immunisation activities [41]. A previous study in Karnataka in 2020 found inadequate knowledge among ASHAs about child immunisation. The above study also documented that by increasing the number of days and focusing on child care the ASHAs had a better understanding of interventions related to child healthcare [42]. As a part of the course structure, ECHO provides one session on new born and post-partum care. An assessment of the number of sessions needed to cover the topics was beyond the scope of our research but would be beneficial.

Previous studies have identified several shortcomings in ASHAs' communication and counselling abilities [43–45]. The findings of this study revealed that the ASHAs faced communication issues while discussing health matters related to family planning and COVID-19 with the community. Previous research has found that interpersonal communication of ASHAs are influenced by factors such as health system support and community context [46]. A study exploring the perspectives of ASHAs on a mobile training course in India also found that they encountered barriers in their interactions with beneficiaries such as resistance from family members, fear of poor quality of care, and financial costs of care [44]. Training programs must therefore, also incorporate how ASHAs can navigate social behaviours and norms to improve the impact of counselling [47, 48]. The extent to which the ECHO training can identify and incorporate community hierarchies to improve communication of the ASHAs needs further exploration. In this study, large batch size (*n =* 40) and limited use of video by participants during the training hampered the engagement between ASHAs as well as with the trainers. A previous study in the USA suggested that limiting batch size and ensuring face-to-face interactions on the virtual platform ensured a higher level of accountability and made it easier to engage with others in the ECHO training sessions [49].

CHWs face significant barriers when using digital technology in LMICs, making it challenging for them to access training on digital platforms [50]. The ASHAs in this study reported an improvement in their ability to use smartphones and navigate mobile applications. Our findings also suggest that ASHAs should be better oriented for accessing content on hand held devices.

The mentorship by trainers added value to participants’ knowledge and helped improve their skills. In this study, participants’ attitudes towards their work changed after attending the ECHO training suggesting that the learning and confidence developed during the training would be transferable to their work in healthcare settings and communities. The ECHO participants of previous studies have also demonstrated similar changes in their practices [35, 40]. Our study findings indicate that the ECHO Model is an effective platform that can help foster a virtual community of practice through case-based learning, shared best practices, and online mentorship by experts.

### Future directions

- There should be more sessions on topics related to post-natal and newborn care as the ASHAs showed poor knowledge and competence in these areas.
- There should be more training on counselling and development of communication skills for ASHAs, specially for maternal and child health and COVID-19.
- An orientation for ASHAs should be conducted to facilitate the use of technology and the platform for learning. This may also help overcome some of the challenges described by the ASHAs in this study.

### Strengths and limitations

The study used a rigorous quasi-experimental design across four different states of India. Our follow-up rate in the study was 80%, indicating a high response from participants completing the pre–post assessment. The presented study has certain limitations. It was not possible to use randomisation and a pure experimental design in this study, and this affects the internal validity of the study. The inclusion of a control group would have strengthened study validity. The self-reported outcomes can be subject to social desirability bias. We did not document the information on attendance and drop outs from the training program. The qualitative results have to be carefully interpreted because of the small sample size of the qualitative study relative to the study sample.

## Conclusion

There is increasing recognition of the importance of CHWs globally for promoting a continuum of care and expanding access to health services. ASHA workers constitute critical human resources in the Indian health system and efforts to empower them are crucial for strengthening the health system at the community level. The encouraging results of this study indicate the effectiveness of Project ECHO in building the capacity of ASHA workers across different geographies in the country.

### Supplementary Information

**Additional file 1: Appendix S1.** **Table S1.1.** Satisfaction with different factors of the training. **Table S1.2.** Satisfaction with content and environment of the training. **Table S1.3.** Challenges faced with respect to ECHO tele-mentoring model. **Table S2.** Technical knowledge and skills. **Table S3.** Statements assessing competence. **Table S4.** Statements assessing attitude and performance.

**Additional file 2: Appendix S2. **Participants in qualitative interviews.

**Additional file 3: Appendix S3.** Key informant Interview Guide for Trainers End line Evaluation.

**Additional file 4: Appendix S4. **Key informant interview guide for Hub leaders End line Evaluation.

**Additional file 5: Appendix S5. **In-depth Interview Guide for ASHAs End line Evaluation.

## Data Availability

All data generated or analyzed during this study are included in this published article (as Additional files).

## Abbreviations

CHW: Community health workers
SDG: Sustainable development goals
NRHM: National Rural Health Mission
ASHA: Accredited social health activists
DIKSHA: Digital infrastructure knowledge sharing
MHRD: Ministry of Human Resource Development
COVID-19: Coronavirus Disease 2019
NHSRC: National Health System Resource Centre
WHO: World Health Organization
HIC: High-Income Countries
IPC: LMICs: Low- and Middle-Income Countries
ECHO: Extension for Community Healthcare Outcomes
IDIs: In-depth interviews
KIIs: Key informant interviews
CME: Continuing medical education
IEC: Institutional Ethics Committee
PIS: Participant Information Sheet
JSPH: Jodhpur School of Public Health
PHFI: Public Health Foundation of India
