# Supplementary material for: A mixed methods evaluation of the impact of ECHO® telementoring model for capacity building of community health workers in India
Source: Hum Resour Health. 2024 Apr 23;22:26. doi: 10.1186/s12960-024-00907-y (PMC11040797; doi:10.1186/s12960-024-00907-y)
Supplement: Supplementary file 1 — Additional file 1: Appendix S1. Table S1.1. Satisfaction with different factors of the training. Table S1.2. Satisfaction with content and environment of the training. Table S1.3. Challenges faced with respect to ECHO tele-mentoring model. Table S2. Technical knowledge and skills. Table S3. Statements assessing competence. Table S4. Statements assessing attitude and performance. [file 12960_2024_907_MOESM1_ESM.docx]

**Appendix 1: Table 1.1. Satisfaction with different factors of the training**

| **Statements towards overall training** | **Response** | **Post ECHO Training N=490** |
| --- | --- | --- |
| The training has added value to your knowledge and experience/skills | Yes | 487 (99.4%) |
|  | No | 3 (0.6%) |
| Was the trainer able to address your concerns regarding field problems? | Yes | 482 (98.4%) |
|  | No | 8 (1.6%) |
| Does the trainer need to share additional training material during the sessions? (Videos, Flipcharts, Reference books) | Yes | 367 (74.9%) |
|  | No | 123 (25.1%) |
| Are you satisfied with the frequency of the training sessions? | Yes | 474 (96.7%) |
|  | No | 16 (3.3%) |
| Was the mode of training accessible? | Yes | 465 (94.9%) |
|  | No | 25 (5.1%) |
| Was training environment conducive to learning (e.g. quiet, clear, uninterrupted) | Yes | 467 (95.3%) |
|  | No | 23 (4.7%) |
| Is there any feedback mechanism of the training? If no, skip the next question | Yes | 400 (81.6%) |
|  | No | 90 (18.4%) |
| Are you satisfied with the feedback mechanism of the training | Yes | 391 (97.8%) |
|  | No | 9 (2.3%) |

**Appendix 1: Table 1.2. Satisfaction with content and environment of the training**

| **Statements towards training content and environment** | **Likert scale Level** | **Post ECHO Training**  **(n=490)** |
| --- | --- | --- |
| Trainers brought relevant field experience to the teaching session | Disagree | 8 (1.6%) |
|  | Neutral | 8 (1.6%) |
|  | Agree | 474 (96.7%) |
| The time allotted for content during each session was adequate. | Disagree | 18 (3.7%) |
|  | Neutral | 11 (2.2%) |
|  | Agree | 461 (94.1%) |
| Sessions were interactive and informative | Disagree | 3 (0.6%) |
|  | Neutral | 4 (0.8%) |
|  | Agree | 483 (98.6%) |
| The case studies, videos & activities were adequate in initiating discussions for replicating real-life scenarios. | Disagree | 8 (1.6%) |
|  | Neutral | 8 (1.6%) |
|  | Agree | 474 (96.7%) |

**Appendix 1: Table 1.3. Challenges faced with respect to ECHO tele-mentoring model**

| **Tele-mentoring factors** | **Level** | **Post ECHO**  **(N = 490)** |
| --- | --- | --- |
| Day of the session | Yes | 63 (12.9%) |
|  | No | 427 (87.1%) |
| Length of Session | Yes | 153 (31.2%) |
|  | No | 337 (68.8%) |
| Frequency of the session | Yes | 153 (31.2%) |
|  | No | 337 (68.8%) |
| Number of people in the session | Yes | 139 (28.4%) |
|  | No | 351 (71.6%) |
| Internet connectivity in the session | Yes | 267 (54.5%) |
|  | No | 223 (45.5%) |

**Appendix 1: Table 2. Technical knowledge and skills**

| **Question** | **Pre-ECHO** | **Post-ECHO** | **P-value** |
| --- | --- | --- | --- |
| Breastfeeding should be started ___ in a normal delivery | 478 (97.6%) | 487 (99.4%) | 0.02 |
| Vignette with case of malaria  What are you suspecting in this case? | 379 (77.3%) | 411 (83.9%) | 0.002 |
| What will you do about the breeding of the mosquitoes? | 431 (88.0%) | 476 (97.1%) | <0.001 |
| Which of the following is not a danger sign in pregnancy? | 287 (58.6%) | 308 (62.9%) | 0.1 |
| Vignette of a case of managing STI  What would you ask her to do? | 473 (96.5%) | 487 (99.4%) | 0.001 |
| Adults should undertake at least ……. minutes of exercise per week. | 226 (46.1%) | 140 (28.6%) | <0.001 |
| Case vignette of a low-birth-weight baby home delivery  How many times would you visit the baby in the first 28 days? | 277 (56.5%) | 255 (52.0%) | 0.1 |
| What schedule (visits) would you follow in the first week? | 5 (1.0%) | 197 (40.2%) | <0.001 |
| Geeta was pregnant for 8 months when she delivered a baby. The baby didn’t breathe, cry or move its limbs at birth. This is: | 283 (57.8%) | 350 (71.4%) | <0.001 |
| What are the vaccinations to be given to the child afterbirth? | 60 (12.2%) | 33 (6.7%) | 0.001 |
| What an ASHA should not do in case of sexual violence: | 260 (53.1%) | 340 (69.4%) | <0.001 |
| How soon after giving birth can a woman undergo sterilization | 212 (43.3%) | 170 (34.7%) | 0.002 |
| A two-year-old child has fast breathing if her breaths per minute are: | 68 (13.9%) | 78 (15.9%) | 0.35 |
| What are the symptoms of COVID-19? | 453 (92.4%) | 457 (93.3%) | 0.58 |
| Which of the following precautions should be undertaken during community visits? | 432 (88.2%) | 424 (86.5%) | 0.39 |
| If nutritional anemia is identified in an adolescent, what services should be provided at the Anganwadi centre? | 275 (56.1%) | 303 (61.8%) | 0.04 |
| Which tablet can you provide for controlling fever and relieving its symptoms? | 489 (99.8%) | 486 (99.2%) | 0.08 |
| What instructions you will give for oral health care? | 211 (43.1%) | 326 (66.5%) | <0.001 |
| Who needs palliative care? | 233 (47.6%) | 344 (70.2%) | <0.001 |
| Which injection would you advise for all persons with wounds to receive at the earliest? | 463 (94.5%) | 469 (95.7%) | 0.3 |

**Appendix 1: Table 3. Statements assessing competence**

| **Competence statements** | **Pre-ECHO**  **(mean ± SD)** | **Post-ECHO (mean ± SD)** | **P-value** |
| --- | --- | --- | --- |
| I feel confident in my ability to screen pregnant women for problems and danger signs and referral | 1.3 ± 1.1 | 1.4 ± 0.7 | 0.01 |
| I feel confident in my ability to identify hypothermia and hyperthermia in newborns. | 1.0 ± 1.3 | 1.1 ± 1.0 | 0.13 |
| I feel confident in my ability to identify birth asphyxia (for home deliveries) and manage with mucus extractor. | 0.2 ± 1.4 | 0.4 ± 1.2 | 0.01 |
| I feel confident in my ability to child immunization tracking skills to ensure complete immunization in the community. | 1.8 ± 0.5 | 1.5 ± 0.7 | <0.001 |
| I feel confident in my ability to help the individual to maintain a healthy blood pressure and control of blood sugar levels by preventing and controlling the risk factors and ensure monthly monitoring of blood pressure and blood sugar. | 1.2 ± 1.1 | 1.3 ± 0.9 | 0.09 |
| I feel confident in my ability to record pregnancy outcomes as abortion, live births, still birth or newborn death. | 1.5 ± 0.9 | 1.4 ± 0.7 | 0.02 |
| I feel confident in my ability to Identifying persons whose symptoms are suggestive of malaria, leprosy, tuberculosis, etc. during home visits, community level care and referral | 1.5 ± 0.8 | 1.4 ± 0.7 | 0.002 |
| I feel confident in my ability to encourage the patients undergoing cancer treatment to have fresh, hygienic, well-cooked foods, avoid alcohol and tobacco in any form. | 1.2 ± 1.0 | 1.3 ± 0.8 | 0.09 |
| I feel confident in my ability to disseminate provisions of acts on domestic violence, sexual harassment etc. | 1.2 ± 1.0 | 1.4± 0.7 | 0.001 |
| I feel confident in my ability to Counsel for post abortion, contraceptive use and for partner treatment in case of STI. | 1.7 ± 0.6 | 1.5 ± 0.7 | <0.001 |
| I feel confident in my ability to monitor symptoms and provide support to the COVID positive patients. | 1.8 ± 0.5 | 1.6 ± 0.5 | <0.001 |
| I feel confident in my ability to clarify the concerns and questions about COVID-19 with the community members | 1.7 ± 0.5 | 1.5 ± 0.6 | <0.001 |

**Appendix 1: Table 4. Statements assessing attitude and performance**

| **Attitude statements** | **Pre-ECHO**  **(mean ± SD)** | **Post-ECHO (mean ± SD)** | **P-value** |
| --- | --- | --- | --- |
| Regular home visits should be given only to low birth weight new born baby | -0.0 ± 1.0 | 0.3 ± 1.0 | <0.001 |
| The family should be involved in the care of the pregnant women | 0.9 ± 0.4 | 1.0 ± 0.3 | 0.009 |
| It is good to isolate HIV patient from community | 0.6 ± 0.8 | 0.7 ± 0.7 | 0.01 |
| I am afraid to become friends with leprosy patients | 0.7 ± 0.7 | 0.7 ± 0.7 | 0.11 |
| It is good not to encourage women in breaking silence about violence. | 0.7 ± 0.6 | 0.8 ± 0.6 | 0.3 |
| Early and exclusive breastfeeding immediately after birth should be discouraged | 0.9 ± 0.5 | 0.6 ± 0.8 | <0.001 |
| It is okay to touch the front surface of the face mask while removing. | 0.8 ± 0.6 | 0.8 ± 0.6 | 0.19 |
