# Supplementary material for: A mixed methods evaluation of the impact of ECHO® telementoring model for capacity building of community health workers in India
Source: Hum Resour Health. 2024 Apr 23;22:26. doi: 10.1186/s12960-024-00907-y (PMC11040797; doi:10.1186/s12960-024-00907-y)
Supplement: Supplementary file 2 — Additional file 2: Appendix S2. Participants in qualitative interviews. [file 12960_2024_907_MOESM2_ESM.docx]

**Appendix 2: Participants in qualitative interviews**

| Participants | Tamil Nadu | Himachal Pradesh | Sikkim | West Bengal | Total (n=21) |
| --- | --- | --- | --- | --- | --- |
| ASHAs | 3 | 3 | 3 | 3 | 12 |
| Trainers | 2 | 1 | 1 | 2 | 6 |
| Hub-leaders | 1 | 1 | 0 | 1 | 3 |
