# Supplementary material for: A mixed methods evaluation of the impact of ECHO® telementoring model for capacity building of community health workers in India
Source: Hum Resour Health. 2024 Apr 23;22:26. doi: 10.1186/s12960-024-00907-y (PMC11040797; doi:10.1186/s12960-024-00907-y)
Supplement: Supplementary file 3 — Additional file 3: Appendix S3. Key informant Interview Guide for Trainers End line Evaluation. [file 12960_2024_907_MOESM3_ESM.docx]

**Appendix 3: Key informant Interview Guide for Trainers**

**End line Evaluation**

**INSTRUCTIONS:**

Consent forms for Key informant Interview participants should be completed before starting the interview by the interviewee. Below is a summary of the information facilitators should use to make sure participant understand the information in the consent form.

**Introduction** (the section below should be read out by the facilitator and ensure that the respondent understand the same).

Thank you for agreeing to participate. We are here to hear about your valuable opinion on the tele-mentoring sessions and support you provide to the ASHAs for enhancing their skills and also about the needs and gaps regarding these trainings.

Explanation of the process: The discussion we are going to have today is called as Key informant Interview.

• Through this discussion we will learn from you about your experiences regarding the trainings that are to be conducted by NHSRC in conjunction with ECHO India.

• You must remember that we’re only trying gather in depth information. This will allow us to understand the context behind the answers and helps us explore solutions in more detail.

Please note

- The interview will last about 30-40 minutes.
- Feel free to ask for any clarification if needed (even in between the discussion)
- This information will be audio recorded with your permission. We will ensure the confidentiality of this recording. The information that you give will be kept confidential and will not be shared by anyone in any manner that can identify you.
- You may stop participating in the interview at any time whenever you wish to.
- In case you decide to withdraw from the study, all information collected from you will be destroyed.

**Turn on the recorder after taking permission to record the interview**

Date of the interview: ………………………………………………

Identification number: ………………………………………………

Name of the participant: ……………………………………………

Questions:

1. Please give a brief description of your roles & responsibilities as a Block/state/national level trainer of ASHAs.
2. What is your experience in conducting these tele mentoring sessions?

Probes:

- Course content & duration
- Teaching technique
- Training Material
- Teaching & learning facilities

1. What are the challenges you face in implementing the tele-mentoring sessions?

Probes:

- Technological barriers
- Interaction level
- Interest of ASHAs

1. Changes observed in the knowledge & skills of the candidates since these sessions are being implemented.

Probes:

- Individual soft skills: Confidence, communication, problem solving etc.
- Knowledge level of ASHAs

1. According to you do these kinds of trainings add any value.

If yes, how?

If no, what is lacking?

1. What according to you are strengths and weaknesses of these sessions?
2. In your opinion, what hinders the participants (ASHA) in implementing learnings from the training sessions in the field?
3. Do you think that these case-based approach will help enhancing the competence, knowledge, and skills of ASHAs?

If yes, how?

If no, why so?

1. Do you think that these tele-mentoring platforms/use of technology will help in enhancing the knowledge and skills of ASHAs in a resource constraint setting like our country?

If yes, how?

If no, why so?

1. What are your suggestions for improving the impact of these tele-mentoring sessions?

That concludes our interview. Thank you so much for sharing your thoughts and opinions with us.

Any specific observation/information related to the interview:
