# Supplementary material for: A mixed methods evaluation of the impact of ECHO® telementoring model for capacity building of community health workers in India
Source: Hum Resour Health. 2024 Apr 23;22:26. doi: 10.1186/s12960-024-00907-y (PMC11040797; doi:10.1186/s12960-024-00907-y)
Supplement: Supplementary file 4 — Additional file 4: Appendix S4. Key informant interview guide for Hub leaders End line Evaluation. [file 12960_2024_907_MOESM4_ESM.docx]

**Appendix 4: Key informant interview guide for Hub leaders**

**End line Evaluation**

**Instructions for the interviewer:**

Consent forms for key informant interview participants should be completed before starting the interview by the interviewee. Below is a summary of the information facilitators should use to make sure participant understand the information in the consent form.

**Introduction** (the section below should be read out by the facilitator and ensure that the respondent understand the same).

Thank you for agreeing to participate. We are here to hear about your valuable opinion on the tele-mentoring sessions and support you provide to the ASHAs for enhancing their skills and also about the needs and gaps regarding these trainings.

Explanation of the process: The discussion we are going to have today is called as key informant Interview.

• Through this discussion we will learn from you about your experiences regarding the trainings that are to be conducted.

• You must remember that we’re only trying gather in depth information. This will allow us to understand the context behind the answers and helps us explore solutions in more detail.

Please note

- The interview will last about 30 to 40 minutes.
- Feel free to ask for any clarification if needed (even in between the discussion)
- This information will be audio recorded with your permission. We will ensure the confidentiality of this recording. The information that you give will be kept confidential and will not be shared by anyone in any manner that can identify you.
- You may stop participating in the interview at any time whenever you wish to.
- In case you decide to withdraw from the study, all information collected from you will be destroyed.

**Turn on the recorder after taking permission to record the interview**

Date of the interview: ………………………………………………

Identification number: ………………………………………………

Name of the participant: ……………………………………………

Questions:

1. Do you think that platforms like ECHO telementoring sessions for trainings of ASHAs are needed?

- If yes, why?
- If no, why?

2. What were your expectations/ goals with the ECHO program? To what extent your expectations/goals have been met?

Probes:

- What are the current needs of ASHAs with regards to training?
- Is ECHO program addressing the current required needs? If yes, How?

3. What is your experience regarding ECHO model?

Probes:

- Case based learning methodology
- Telementoring
- Frequency of trainings

4. What are the challenges and barriers that you have experienced in using ECHO program for ASHAs training?

Probes:

- Which components of the programs are facing the challenges? What are the immediate corrective measures you have undertaken to resolve these challenges?
- The major areas that needs strengthening
- Any administrative/logistical issues at your Hub
- Support from ECHO
- Coordination with other stakeholders of the program
- Reporting process
- Overcome the hierarchal structure for active participation of ASHAs

5. Changes observed in the knowledge & skills of the candidates since these sessions are being implemented?

Probes:

- Personal Soft skills: Confidence, communication, problem solving etc.
- Knowledge level of ASHAs

6. Please tell us the uniqueness you have experienced by using ECHO program for ASHAs training.

7. Do you think that it is possible to sustain and scale up the ECHO model for ASHAs training?

If yes, how would like to scale up/ expand your ECHO Programme?

If no, what do you think are the barriers in scaling up the program?

8. Are you getting enough support and motivation to implement ECHO model in your area from your immediate leadership?

If yes, what are the aspects of the program in which you are getting support?

If no, in what areas do you need the support?

9. Any other thing you want to add regarding ASHA trainings?

That concludes our interview. Thank you so much for sharing your thoughts and opinions with us.

Any specific observation/information related to the interview:
