# Supplementary material for: A mixed methods evaluation of the impact of ECHO® telementoring model for capacity building of community health workers in India
Source: Hum Resour Health. 2024 Apr 23;22:26. doi: 10.1186/s12960-024-00907-y (PMC11040797; doi:10.1186/s12960-024-00907-y)
Supplement: Supplementary file 5 — Additional file 5: Appendix S5. In-depth Interview Guide for ASHAs End line Evaluation. [file 12960_2024_907_MOESM5_ESM.docx]

**Appendix 5: In-depth Interview Guide for ASHAs**

**End line Evaluation**

**Instructions for the interviewer:**

Consent forms for in-depth interview participants should be completed in advance by all those seeking to participate. Below is a summary of the information facilitators should use to make sure participants understand the information in the consent form.

**Introduction** (the section below should be read out by the facilitator and ensure that the participants understand the same).

Thank you for agreeing to participate. We are here to hear about your valuable opinion on the training and support provided to you for enhancing your skills as an ASHA and about the needs and gaps regarding these training.

Explanation of the process: The discussion we are going to have today is called an in-depth interview.

- Through this discussion, we would like to learn about your experiences on training through the telementoring platform.
- You must remember that we are only gathering in-depth information.
- This will allow us to understand the context behind the answers and helps us to explore alternatives distinctively.

Please note

- In-depth interview will last about 30-40 minutes.
- Feel free to ask for any clarification if needed (even in between the discussion)
- This information will be audio recorded with your permission. We will ensure the confidentiality of this recording. The information that you give will be kept confidential and will not be shared by anyone in any manner that can identify you.
- You may stop participating in the interview at any time whenever you wish to.
- In case you decide to withdraw from the study, all information collected from you will be destroyed.

**Turn on the recorder after taking permission to record the interview**

Date of the interview: ………………………………………………

Identification number: ………………………………………………

Name of the participant: ……………………………………………

Make sure to give participants time to think before answering the questions and don’t move too quickly. Use the probes to make sure that all issues are addressed but move on when you feel you are starting to hear repetitive information.

Questions:

1) Let us start the interview by talking about your roles and responsibilities as ASHAs.

2) Please describe a normal day at work for you.

Probes:

- Activities and duties performed
- Challenges faced at work

3) Why did you participate in these telementoring sessions?

Probes:

- Source of information
- Motivation/need to join the program
- Expectations from the program

4) Please share your experience regarding the telementoring sessions that are being conducted for you.

Probes:

- Benefits
- Challenges – Frequency of training, logistics
- Teaching Methods and trainers
- Topics covered
- Mode of Training- Comfort with online training- if preference for offline then,

What component of the offline training can be included and how?

- ECHO technology -video conference, presentations, audio

6) Let us discuss the environment of training sessions now.

Probes:

- Comfort in asking doubts/questions with the trainer
- Views about the peer learning (learning from/with fellow ASHA workers)

7) Let us discuss your opinion regarding the topics being covered through telementoring sessions. Ask about the following topics one by one:

Probes:

• Selection of the topics- appropriate, useful for their work

• Extent of coverage of the topics in the training/course curriculum

• Time given to a specific topic

- Example of a topic that was covered

8) Do you think that the case-based approach of these tele mentoring sessions are beneficial to you?

If yes, in what ways?

If no, what is lacking?

Probes:

- Who presents the case
- When are you informed regarding the case presentation?
- Do you get adequate time to prepare?
- How do you prepare?
- How do you present the case?
- Discussion after presentation- adequate, thoughts for improvement

9) Kindly share an example where you have been able to implement the skills/knowledge provided by these training in your work field.

10) What changes (if any) you observe in yourselves after undergoing these telementoring sessions?

Probes:

- Confidence
- Knowledge & Skills
- Attitude
- Practices

11) Please provide your suggestions for improving the quality and utility of these sessions.

Probes:

- Teaching methods
- Topic selection
- Teaching material
- Training environment

12) Please specify the areas of your job in which you would like to receive further training or support.

13) If such training, are to be provided in the future at your Sub centres without any monetary incentives, will you be attending those?

If yes, what are your aspirations to attend those?

If no, why?

Probes:

- Enhancement in learning/knowledge
- Ease of learning
- Comfortability with the online platform

14) Is there anything else you would like to add regarding telementoring ASHA’s training?

That concludes our interview. Thank you so much for sharing your thoughts and opinions with us.

Any specific observation/information related to the IDI:
